# Clinical Evaluation of *Ganoderma lucidum* Spore Oil for Triglyceride Reduction: A Randomized, Double-Blind, Crossover Study

**DOI:** 10.3390/nu17050844

**Published:** 2025-02-28

**Authors:** Xinyi Wang, Xufeng Wang, Liang Zhao, Feng Zhou

**Affiliations:** 1 Beijing Key Laboratory of Functional Food from Plant Resources, College of Food Science and Nutritional Engineering, China Agricultural University, Beijing 100083, China; xaesi23@163.com; 2 Capital Healthcare and Nutrition Cuisine Society, Beijing 100082, China; 18801300305@163.com; 3 Key Laboratory of Geriatric Nutrition and Health, Beijing Technology and Business University, Ministry of Education, Beijing 100048, China; liangzhao@btbu.edu.cn; 4 China Agricultura University-Sichuan Advanced Agricultural & Industrial Institute, China Agricultural University, Chengdu 611430, China

**Keywords:** *Ganoderma lucidum* spore oil, dyslipidemia, clinical applications

## Abstract

**Background**: *Ganoderma lucidum* spore oil (GLSO) is widely recognized for its notable medicinal and nutritional properties. This study aimed to systematically evaluate the efficacy and safety of GLSO extract in individuals with dyslipidemia. **Methods**: In a randomized, double-blind, placebo-controlled trial, 110 participants were enrolled and randomly assigned to either the intervention group or the placebo group. A chi-square test of baseline characteristics confirmed no significant differences in age or sex distribution between the two groups. **Results**: After 12 weeks of intervention, the intervention group exhibited significantly lower levels of total cholesterol (CHO), triglycerides (TG), and low-density lipoprotein cholesterol (LDL-C), alongside significantly higher levels of high-density lipoprotein cholesterol (HDL-C), compared to the placebo group, with all differences reaching statistical significance. Furthermore, the relative percentage changes in lipid parameters also demonstrated significant intergroup differences. Safety analyses revealed that the intervention had no notable effects on renal function parameters, whereas hepatic function parameters showed statistically significant improvement in the intervention group. **Conclusions**: This study demonstrated that GLSO extract effectively improved lipid profiles and liver function, with a favorable safety and tolerability profile. These findings strongly support the potential clinical application of GLSO extract in the management of dyslipidemia.

## 1. Introduction

*Ganoderma lucidum* is a fungus belonging to the *Ganoderma* genus, renowned for its significant medicinal and nutritional value [1]. The spores of *Ganoderma lucidum* are ovoid germ cells released from the folds of the fruiting body and have been shown to exert diverse biological effects, including antitumor activity, hepatoprotection, and immune modulation [2]. *Ganoderma lucidum* spore oil (GLSO) is a lipid-soluble extract derived from these spores, enriched with bioactive compounds such as terpenoids, sterols, unsaturated fatty acids, and polysaccharides [3]. Triterpenoid compounds are the main bioactive components of GLSO. Studies have shown that the content of triterpenoids in GLSO is significantly higher than that in *Ganoderma lucidum* spore powder (GLS) [4]. Due to the highly lipophilic nature of triterpenoids, their extraction from GLSO is challenging, which has resulted in limited research on the isolation and identification of triterpenoids in GLSO [5]. GLSO has been demonstrated to possess immunomodulatory, hepatoprotective, lipid-regulating, and antitumor properties [6,7]. However, studies on the mechanism by which GLSO regulates lipid metabolism remain limited. Some literature suggests that GLSO may modulate the expression of lipid metabolism-related proteins, such as uncoupling protein 1 (Ucp1), myelin protein zero (Mpz), and fatty acid synthase (Fasn), by regulating the gut microbiota and maintaining metabolic stability in the heart [8]. It has been demonstrated that GLSO can alleviate hepatic dysfunction and reduce inflammatory responses by modulating lipid metabolic pathways associated with glycerophospholipid and sphingolipid metabolism. The underlying mechanisms include a reduction in the levels of lysophosphatidylcholine (LPC), glycerophosphatidylcholine (GPC), and sphingosine 1-phosphate (S1P), alongside an increase in the levels of phosphatidylglycerol (PG) and galactosylceramide (GGC) [9]. Furthermore, GLSO is characterized by excellent stability and safety, making it suitable for clinical and dietary use. It is commonly administered in capsule form, leveraging its broad range of applications [10].

The sedentary lifestyle and unhealthy dietary habits prevalent in modern society have contributed to a steady annual increase in the prevalence of dyslipidemia, with a notable trend toward younger populations [11]. Elevated triglycerides (TG) are a mark of dyslipidemia [12], which is well established as a significant risk factor for the development of cardiovascular diseases (CVDs) [13]. CVDs represent a major contributor to the global disease burden, accounting for tens of millions of deaths annually, according to the European Society of Cardiology’s 2023 Statistical Atlas of Cardiovascular Diseases, and the treatment of CVDs constitutes 11% of total healthcare expenditures in the European Union, underscoring the substantial economic and social burden they impose [14]. Studies have demonstrated that effective reduction in TG levels can significantly slow the progression of cardiovascular diseases [15]. Furthermore, elevated TG levels are strongly associated with an increased risk of developing non-alcoholic fatty liver disease (NAFLD) [16]. Similarly, elevated TG levels have been shown to negatively affect blood pressure and are recognized as an independent risk factor for the development of hypertension [17]. Currently, fenofibrate is a commonly used fibrate drug for the treatment of hypertriglyceridemia and hypercholesterolemia. However, studies have shown that approximately 20% of patients may experience elevated serum aminotransferase levels while using fenofibrate, and in some cases, this can progress to chronic liver injury [18]. Additionally, experimental data have demonstrated that fenofibrate effectively reduces TG levels in young rats without significantly affecting their alkaline phosphatase (ALP) levels. In contrast, in older rats treated with fenofibrate, serum ALP levels were significantly elevated, and liver structure was markedly impaired [19].

Functional constituents derived from plants have emerged as a key focus in modern research aimed at developing novel hypolipidemic agents. This interest stems from their diverse biological activities, natural origin, and minimal toxic side effects. Recent studies have demonstrated that various plant extracts and derivatives exhibit significant adjuvant hypolipidemic effects. Examples include rosemary extract [20], hempseed peptides [21], and rapeseed protein-derived peptides [22], all of which have shown promising potential in lipid regulation.

In conclusion, GLSO extract shows potential lipid-lowering effects. However, existing studies primarily focus on preclinical animal models, with a notable lack of systematic clinical validation in human populations. Chu et al. demonstrated the beneficial effect of *Ganoderma lucidum* supplementation in improving dyslipidemia in diabetic patients through a clinical trial. However, its effects on other populations still require further investigation and validation [23]. As a result, the aim of this study is to evaluate the effect of GLSO on lipid levels in individuals with elevated triglyceride levels through a clinical trial. Additionally, this study will provide a comprehensive evaluation of the long-term safety of GLSO, with particular emphasis on its effects on liver and kidney functions. The findings will offer empirical evidence to support the clinical application, promotion, and further scientific development of GLSO.

## 2. Materials and Methods

### 2.1. Test Samples

GLSO extracts were administered to the study participants in the form of softgel capsules. The GLSO extract (which contains 33.7 g of triterpenoid compounds per 100 g) used in this study were purchased by Shanghai Kendao Industrial Co., Ltd. (Shanghai, China). The researchers instructed each participant to take 3 softgel capsules per dose, once in the morning and once in the evening each day, with each softgel containing 0.5 g of the extract. The daily intake of GLSO in the intervention group was calculated to be approximately 3.0 g, corresponding to an intake of approximately 1.011 g of total triterpenes. It has been shown that the safe dose of GLSO should not exceed 0.42 g/(kg·d) [24]. Based on this criterion, the GLSO dose used in the trial was well below the recommended upper safety limit for normal adults and, therefore, met the requirements for safe use. The placebo group received identical placebo capsules, which were indistinguishable in appearance from the active treatment capsules.

### 2.2. Population Recruitment

This experiment was designed based on the revised guidelines by the State Food and Drug Administration of China, which served as the guiding reference for the principles of population recruitment. This study was conducted between July and October 2024 and involved a total of 113 participants, aged 18 to 65 years, with TG levels between 1.70 and 2.25 mmol/L. Before the trial commenced, all participants were provided with an informed consent form, ensuring that each participant voluntarily agreed to take part in this study. To maintain the integrity and accuracy of the trial results, participants were explicitly instructed not to use any lipid-lowering supplements or medications throughout the duration of this study. Participants were randomly assigned to 10 groups, with each group designated a liaison officer. The liaison officer was responsible for recording participants’ basic demographic information, including name, sex, age, and long-term medication history. Throughout this study, each liaison officer monitored and documented the medication usage and daily status of participants at regular intervals (morning and evening), ensuring that no participant experienced any significant abnormalities or adverse events during the trial period. Participants who met any of the following exclusion criteria were not included in this study: age younger than 18 or older than 65 years; pregnancy or breastfeeding; known allergies or hypersensitivity to any of the test samples; severe cardiac, hepatic, renal, or hematopoietic disorders, or any psychiatric conditions; use of lipid-lowering medications within the past two weeks that could influence the outcome measures; hospitalization due to high blood cholesterol levels; failure to consume the test samples as instructed; or incomplete information that could affect the assessment of efficacy or safety.

This study was approved by the Ethics Committee of Beijing Key Laboratory of Functional Foods from Plant Resources (License No. A330-2024-9).

### 2.3. Experimental Methods

This study employed a double-blind, randomized, placebo-controlled design to evaluate the efficacy of GLSO extract in reducing blood lipid levels in an adult population. Prior to the commencement of the trial, all participants underwent baseline blood tests at a designated hospital (Shijiazhuang, China). Based on the key indicators from the blood test results, this study employed a stratified randomization method to categorize participants according to their lipid levels. Participants were then randomly assigned to either the intervention group or the placebo group using computer-generated random numbers. To ensure that each group contained at least 50 participants, the randomization process was fully computerized, and all group assignment information was kept strictly confidential. To ensure comparability between groups, chi-square tests were conducted on baseline variables (e.g., age and sex) and baseline hematological variables that could potentially influence the study outcomes.

The primary indicators for assessing the lipid-lowering effect included total cholesterol (TC), low-density lipoprotein cholesterol (LDL-C), high-density lipoprotein cholesterol (HDL-C), and TG. To evaluate the safety profile, additional biomarkers were measured to assess liver, kidney, and biliary function, including total bilirubin, direct bilirubin, alanine aminotransferase (ALT), aspartate aminotransferase (AST), γ-glutamyl transferase (GGT), total bile acids (TBA), cholinesterase, ALP, calcium (Ca), urea, uric acid, chloride (Cl), sodium (Na), potassium (K), and creatinine (Cr). All biomarkers were measured at two time points: once before the intervention began and once at the end of the study.

### 2.4. Statistical Analysis

The data management and statistical analysis for this study strictly followed the guidelines by the State Food and Drug Administration of China. Once the health examination reports for all participants were generated, the research team created a series of restricted-access files and sent them to the statisticians, ensuring strict confidentiality of the participants’ data. The results of this study are presented as means ± standard deviation (SD). Chi-square tests were used to evaluate the baseline differences of participants’ data (e.g., age and sex). When the *p*-value was greater than 0.05, the baseline characteristics between the two groups were considered not to differ significantly. The significance of differences between groups was analyzed using paired *t*-tests and independent samples *t*-tests. Differences were considered statistically significant when the *p*-value was less than 0.05. All statistical analyses were performed using SPSS version 26.0 (IBM/SPSS Corp., Chicago, IL, USA), and graphical representations were generated using GraphPad Prism version 8.0 (GraphPad Software, San Diego, CA, USA).

## 3. Results

### 3.1. Baseline Analysis

**Figure 1:**
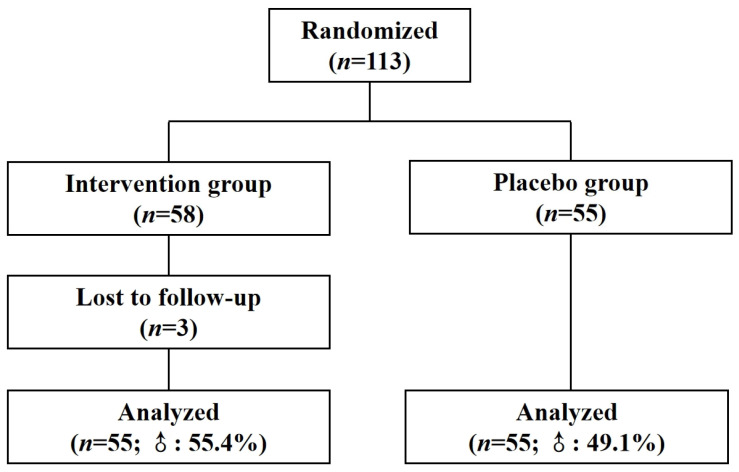
Diagram illustrating the flow of participants and key stages in the current study.

A total of 113 participants were recruited and randomly assigned to either the intervention group (*n* = 58) or the placebo group (*n* = 55). During the trial, three participants from the intervention group withdrew, leaving 55 participants in each group for final analysis. The mean ages in the intervention and placebo groups were 55.22 ± 13.58 and 57.55 ± 8.41 years, respectively. There was no significant difference in sex distribution between groups (55.4% male in the intervention group vs. 49.1% male in the placebo group; *p* = 0.509, chi-square test). Baseline comparisons revealed no significant differences in TG, TC, LDL-C, and HDL-C level between the two groups (*p* > 0.05), suggesting that the groups were comparable at baseline. Detailed data are presented in Table 1. The flowchart illustrating the progression of this study is presented in Figure 1.

### 3.2. Lipid Parameters Analysis

As shown in Table 2, after 12 weeks of treatment, the intervention group exhibited significantly lower levels of TG (1.51 ± 0.55 mmol/L), LDL-C (2.68 ± 0.67 mmol/L), and TC (4.87 ± 0.89 mmol/L) compared to the placebo group (TG: 1.88 ± 0.16 mmol/L, LDL-C: 3.20 ± 0.97 mmol/L, TC: 5.25 ± 0.91 mmol/L; *p* < 0.05). Conversely, HDL-C levels were significantly higher in the intervention group (1.51 ± 0.22 mmol/L) than in the placebo group (1.36 ± 0.30 mmol/L; *p* < 0.05).

**Figure 2:**
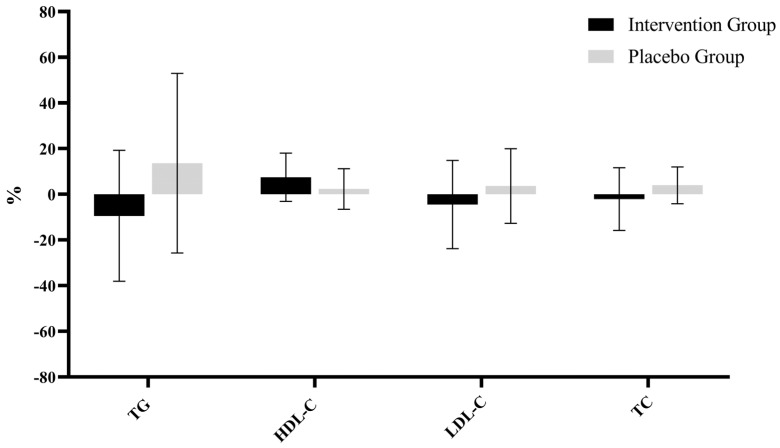
Relative percentage changes in lipid outcomes between the intervention and placebo groups. TG: triglycerides, TC: total cholesterol, LDL-C: low-density lipoprotein cholesterol, HDL-C: high-density lipoprotein cholesterol.

As shown in Figure 2, significant differences were observed in the relative percentage changes in lipid indices before and after the trial between the intervention and placebo groups. Specifically, the intervention group demonstrated a relative decrease in TG of −22.18 ± 27.87%, which was significantly lower than the −1.98 ± 8.45% observed in the placebo group (*p* < 0.001). The relative change in HDL-C was 7.44 ± 0.46% in the intervention group, significantly higher than the 2.40 ± 8.87% in the placebo group (*p* = 0.019). For LDL-C, the intervention group showed a relative decrease of −4.1 ± 19.14%, significantly lower than the 3.6 ± 16.34% increase in the placebo group (*p* = 0.038). Similarly, the relative change in TC was −1.98 ± 13.62% in the intervention group, significantly lower than the 4.01 ± 8.02% change in the placebo group (*p* = 0.011).

### 3.3. Safety Parameters Analysis

As shown in Table 3, among the safety parameters, the intervention group exhibited significantly lower levels of ALP (74.94 ± 12.68 U/L), TBA (3.20 ± 1.47 µmol/L), AST (21.00 ± 5.37 U/L), ALT (20.94 ± 6.52 U/L), GGT (23.19 ± 14.60 U/L), direct bilirubin (3.43 ± 1.12 µmol/L), and total bilirubin (13.00 ± 4.10 µmol/L) compared to the placebo group (ALP: 85.20 ± 24.57 U/L, TBA: 3.95 ± 1.92 µmol/L, AST: 24.17 ± 6.04 U/L, ALT: 33.13 ± 24.80 U/L, GGT: 4.20 ± 1.14 µmol/L, direct bilirubin: 25.69 ± 11.81 µmol/L, total bilirubin: 15.83 ± 5.35 µmol/L; *p* < 0.05). Additionally, cholinesterase levels were significantly higher in the intervention group (10.27 ± 1.45 kU/L) compared to the placebo group (9.21 ± 1.29 kU/L; *p* < 0.01), suggesting that GLSO extract may improve hepatobiliary function at the population level.

No significant differences were observed between the intervention and placebo groups for renal function markers, including Cr, K, Na, Cl, uric acid, urea, and Ca (*p* > 0.05), indicating that the GLSO extract does not have toxic effects on the liver or kidneys.

## 4. Discussion

GLSO is an active compound extracted from the spores of *Ganoderma lucidum* (Leyss. ex Fr) Carstairs, known for its significant antioxidant, anti-inflammatory, and immunomodulatory properties. The primary components of GLSO include triterpenoids and unsaturated fatty acids [25,26]. While research on the effects of GLSO in metabolic diseases is still limited, available studies indicate its potential in modulating lipid metabolism. For example, Li et al. demonstrated that GLSO, rich in unsaturated fatty acids and triterpenoids, effectively reduced serum TC and LDL-C levels and alleviated lipid deposition in hyperlipidemic New Zealand rabbits [27]. Additionally, Li et al. examined the hepatoprotective effects of GLSO and found that its administration significantly decreased the levels of AST and ALT in a carbon tetrachloride (CCl4)-induced chemical liver injury model in mice [28]. The clinical study conducted in this trial demonstrated that GLSO exhibits a significant lipid-lowering effect in individuals with borderline high TG levels. Specifically, GLSO effectively reduced TC, TG, and LDL-C levels while increasing HDL-C levels. Furthermore, in addition to its role in lipid metabolism regulation, GLSO also led to a reduction in AST and ALT levels, indicating a potential hepatoprotective effect. These findings align with previous animal studies, further supporting the lipid-lowering and hepatoprotective potential of GLSO in clinical applications.

When TG levels are elevated, they can be classified into three categories based on their concentration: borderline high (1.69–2.25 mmol/L), high (2.25–5.63 mmol/L), and very high (≥5.64 mmol/L) [29]. This study focuses on the lipid-lowering effect of GLSO in individuals with TG levels in the borderline high range. Numerous studies have shown that elevated TG levels are positively associated with an increased risk of cardiovascular disease (CVD) events. However, most current medications for the treatment of CVDs primarily focus on statins. Although statins can reduce TG levels to some extent, their main effect is still focused on lowering LDL-C levels [30]. Fibrates act as peroxisome proliferator-activated receptor alpha (PPARα) agonists, primarily by activating PPARα. Studies have shown that fibrates reduce TG levels and increase HDL-C levels [31]. However, the results of a study by Kim et al. suggest that when fibrates are used as adjuncts to statins in populations with elevated TG levels, they may require more than one year of treatment to show an effect on CVD. Thus, while fibrates play a role in lowering TG and increasing HDL-C, their impact on improving cardiovascular outcomes may take a longer duration of treatment to become evident [32]. All in all, for individuals with borderline high TG levels, it is important to identify functional foods that can assist in lowering blood lipids, thereby reducing the potential side effects and risks associated with drug therapy.

It has been demonstrated that the novel yellow pigment monascinol (Msol) in red mold rice can inhibit fatty acid synthesis by activating acetyl-CoA carboxylase (ACC), thereby reducing TG accumulation [33]. Additionally, plant sterol supplementation has been shown to significantly reduce TG levels in hypertriglyceridemic individuals, potentially by interrupting fatty acid absorption in the gut [29]. Both plant sterols and monascinol from red mold rice have been shown to significantly lower blood TG levels in individuals with hypertriglyceridemia by modulating lipid metabolic pathways. In comparison to these findings, the results from studies involving GLSO at the population level also indicate a similar effect, demonstrating its potential as an adjunctive lipid-lowering agent in individuals with elevated TG levels. This effect likely occurs through the inhibition of fat accumulation and the modulation of lipid metabolism-related pathways. However, further investigation is needed to clarify the specific mechanisms underlying the lipid-lowering effects of GLSO, which should be validated through animal models and cellular studies.

Therefore, the results of this experiment showed that long-term use of GLSO-containing softgel capsules significantly reduced TG levels in individuals with elevated TG levels. Additionally, it had a significant lowering effect on LDL-C and TC levels, while effectively increasing HDL-C levels. The difference in lipid levels between the intervention group and the placebo group was statistically significant (*p* < 0.05). Therefore, GLSO may be considered as an adjunctive therapy to statins and fibrates for patients requiring additional control of TG levels, or as a nutritional supplement for populations with borderline high TG levels. Regarding renal function assessment, no significant differences were observed between the intervention and placebo groups in terms of Cr, K, Na, Cl, uric acid, urea, and Ca levels (*p* > 0.05), indicating that GLSO extracts did not induce toxic effects on the kidneys. Furthermore, the relative percentage changes in safety indices between the intervention and placebo groups revealed significant differences for ALP, cholinesterase, TBA, aspartate aminotransferase (AST), gamma-glutamyl transferase (GGT), direct bilirubin, alanine aminotransferase (ALT), and TBIL (*p* < 0.05). These results further support the potential beneficial effects of GLSO extract on hepatobiliary function.

Notably, in the analysis of pre- and post-treatment differences in safety indicators, significant differences were observed, particularly in the levels of ALP and ALT in the placebo group. This result suggests that individual participant variability, psychological state, compliance, and the relatively short duration of the trial may have contributed to these differences. Therefore, future studies should implement more stringent data screening procedures and apply appropriate statistical adjustments to better handle outliers, thereby ensuring the robustness and reliability of the findings.

Overall, the intervention group showed significant improvements in hepatobiliary function, while no significant differences were observed between the intervention and placebo groups in terms of renal function, suggesting that the GLSO extract is not significantly toxic with respect to liver and renal safety.

In summary, this study provides preliminary evidence that GLSO extract has potential lipid-lowering effects and demonstrates a favorable safety profile. However, the participants in this trial were focused on individuals with borderline high TG levels, and the lack of evaluation of individuals with high triglyceride levels may somewhat limit the broad applicability of the trial results. In addition, although this trial lasted only 12 weeks, *Ganoderma lucidum* has been included in the *Chinese Pharmacopoeia*, which was jointly developed and published by the State Council’s administrative department of health and the State Council’s department of food safety supervision and management. This inclusion serves as strong evidence of its safety. Furthermore, Xia et al. conducted an experiment in which they administered *Ganoderma lucidum* spore powder at a dose 20 times higher than the human equivalent in a 26-week gavage study on both female and male rats. The results indicated that *Ganoderma lucidum* spore powder did not cause any adverse effects in the rats, further supporting its safety [34]. Based on these findings, we believe that even with long-term use of GLSO, the likelihood of adverse effects on humans remains low. However, future studies should include longer follow-up periods to more thoroughly assess its long-term effects and safety. On the other hand, this trial primarily assessed the efficacy of GLSO extract in lowering blood lipids in individuals with borderline high triglyceride levels at the clinical level; however, it lacked an in-depth exploration of its mechanism of action. Therefore, more systematic and comprehensive mechanistic studies should be conducted in the future to clarify the specific biological mechanisms of GLSO in lowering blood lipids.

## 5. Conclusions

The results of this study indicate that GLSO extract effectively improved dyslipidemia in individuals with borderline high TG levels, as demonstrated by reductions in TG, TC, and LDL-C levels, along with an increase in HDL-C levels. After a 12-week trial, GLSO extract showed no adverse effects on renal function in any of the participants and also contributed to improvements in liver function, further supporting the safety of GLSO extract. These findings suggest that GLSO extract holds potential as a dietary supplement to address lipid metabolism disorders, particularly for individuals with dyslipidemia or those at high risk for cardiovascular disease.

While this study provides strong evidence that GLSO extract regulates lipid metabolism at the clinical level, the specific molecular mechanisms underlying its effects remain unclear. Further in-depth research is required to fully elucidate the mechanisms of action of GLSO extract.

## Figures and Tables

**Table 1:** Comprehensive baseline analysis of lipid profiles, including TG, TC, LDL-C, and HDL-C, in the intervention and placebo groups.

Parameter	Intervention Group (*n* = 58)	Placebo Group (*n* = 55)	*p-*Value	Analysis of Intergroup Statistical Differences
	Mean ± SD	Mean ± SD		
TG (mmol/L)	1.94 ± 0.17	1.93 ± 0.17	0.791	*p* > 0.05
HDL-C (mmol/L)	1.41 ± 0.22	1.33 ± 0.31	0.164	
LDL-C (mmol/L)	2.87 ± 0.79	3.08 ± 0.78	0.200	
TC (mmol/L)	5.02 ± 0.93	5.06 ± 0.85	0.824	

TG: triglycerides, TC: total cholesterol, LDL-C: low-density lipoprotein cholesterol, HDL-C: high-density lipoprotein cholesterol. The *p*-value was calculated using an independent samples *t*-test. Differences between groups were considered statistically significant when *p* < 0.05.

**Table 2:** Lipid outcomes after 12 weeks of intervention or placebo treatment, comparing changes in lipid levels by group.

Parameter	Intervention Group (*n* = 55)	Placebo Group (*n* = 55)	*p-*Value
	Mean ± SD	Mean ± SD	
TG (mmol/L)	1.51 ± 0.55	1.88 ± 0.16	<0.001
HDL-C (mmol/L)	1.51 ± 0.22	1.36 ± 0.30	0.011
LDL-C (mmol/L)	2.68 ± 0.67	3.20 ± 0.97	0.004
TC (mmol/L)	4.87 ± 0.89	5.25 ± 0.91	0.041

TG: triglycerides, TC: total cholesterol, LDL-C: low-density lipoprotein cholesterol, HDL-C: high-density lipoprotein cholesterol. The *p*-value was calculated using an independent samples *t*-test. Differences between groups were considered statistically significant when *p* < 0.05.

**Table 3:** Safety outcomes by group after 12 weeks of intervention or placebo treatment.

Parameter	Intervention Group (*n* = 55)	Placebo Group (*n* = 55)	*p*-Value
	Mean ± SD	Mean ± SD	
ALP (U/L)	74.94 ± 12.68	85.20 ± 24.57	0.032
cholinesterase (kU/L)	10.27 ± 1.45	9.21 ± 1.29	0.001
TBA (µmol/L)	3.20 ± 1.47	3.95 ± 1.92	0.047
AST (U/L)	21.00 ± 5.37	24.17 ± 6.04	0.011
GGT (U/L)	23.19 ± 14.60	33.13 ± 24.80	0.020
direct bilirubin (µmol/L)	3.43 ± 1.12	4.20 ± 1.14	0.003
ALT (U/L)	20.94 ± 6.52	25.69 ± 11.81	0.041
total bilirubin (µmol/L)	13.00 ± 4.10	15.83 ± 5.35	0.008
Cr (µmol/L)	63.00 ± 12.18	63.64 ± 13.25	0.799
K (mmol/L)	4.07 ± 0.29	4.01 ± 0.32	0.360
Na (mmol/L)	143.50 ± 1.65	143.69 ± 1.52	0.516
Cl (mmol/L)	104.89 ± 2.01	105.46 ± 1.71	0.117
uric acid (µmol/L)	328.76 ± 80.01	322.83 ± 84.45	0.731
urea (mmol/L)	5.24 ± 1.41	4.92 ± 1.27	0.229
Ca (mmol/L)	2.40 ± 0.05	2.41 ± 0.09	0.398

ALT: alanine aminotransferase, AST: aspartate aminotransferase, GGT: γ-glutamyl transferase, TBA: total bile acids, ALP: alkaline phosphatase, Ca: calcium, Cl: chloride, Na: sodium, K: potassium, Cr: creatinine. The *p*-value was calculated using an independent samples *t*-test. Differences between groups were considered statistically significant when *p* < 0.05.

## Data Availability

The datasets generated and/or analyzed during the current study are not publicly available to protect patient confidentiality; however, these are available from the corresponding author upon reasonable request.
